# Ultrasound-Based Radiomics Analysis for Preoperatively Predicting Different Histopathological Subtypes of Primary Liver Cancer

**DOI:** 10.3389/fonc.2020.01646

**Published:** 2020-09-24

**Authors:** Yuting Peng, Peng Lin, Linyong Wu, Da Wan, Yujia Zhao, Li Liang, Xiaoyu Ma, Hui Qin, Yichen Liu, Xin Li, Xinrong Wang, Yun He, Hong Yang

**Affiliations:** ^1^Department of Medical Ultrasonics, The First Affiliated Hospital of Guangxi Medical University, Nanning, China; ^2^GE Healthcare, Shanghai, China

**Keywords:** primary liver cancer, histopathological subtype, radiomics, ultrasound, identification

## Abstract

**Background:**

Preoperative identification of hepatocellular carcinoma (HCC), combined hepatocellular–cholangiocarcinoma (cHCC-ICC), and intrahepatic cholangiocarcinoma (ICC) is essential for treatment decision making. We aimed to use ultrasound-based radiomics analysis to non-invasively distinguish histopathological subtypes of primary liver cancer (PLC) before surgery.

**Methods:**

We retrospectively analyzed ultrasound images of 668 PLC patients, comprising 531 HCC patients, 48 cHCC-ICC patients, and 89 ICC patients. The boundary of a tumor was manually determined on the largest imaging slice of the ultrasound medicine image by ITK-SNAP software (version 3.8.0), and then, the high-throughput radiomics features were extracted from the obtained region of interest (ROI) of the tumor. The combination of different dimension-reduction technologies and machine learning approaches was used to identify important features and develop the moderate radiomics model. The comprehensive ability of the radiomics model can be evaluated by the area under the receiver operating characteristic curve (AUC).

**Results:**

After digitally processing tumor ultrasound images, 5,234 high-throughput radiomics features were obtained. We used the Spearman + least absolute shrinkage and selection operator (LASSO) regression method for feature selection and logistics regression for modeling to develop the HCC-vs-non-HCC radiomics model (composed of 16 features). The Spearman + statistical test + random forest methods were used for feature selection, and logistics regression was applied for modeling to develop the ICC-vs-cHCC-ICC radiomics model (composed of 19 features). The overall performance of the radiomics model in identifying different histopathological types of PLC was moderate, with AUC values of 0.854 (training cohort) and 0.775 (test cohort) in the HCC-vs-non-HCC radiomics model and 0.920 (training cohort) and 0.728 (test cohort) in the ICC-vs-cHCC-ICC radiomics model.

**Conclusion:**

Ultrasound-based radiomics models can help distinguish histopathological subtypes of PLC and provide effective clinical decision making for the accurate diagnosis and treatment of PLC.

## Introduction

Primary liver cancer (PLC) is one of the most lethal and prevailing tumors, which is estimated to rank the fifth in cancer mortality among men and the seventh among women. In recent years, the incidence of PLC has continued to increase, rising faster than that of other cancers (1, 2). In the same solid malignant neoplasm, PLC can be classified according to histological sources. A tumor that contains only cancerous hepatocytes is defined as hepatocellular carcinoma (HCC), only cancerous bile duct cells are defined as intrahepatic cholangiocarcinoma (ICC), and a mixture of HCC and ICC is defined as combined hepatocellular–cholangiocarcinoma (cHCC-ICC) (3, 4).

cHCC-ICC is a relatively rare subtype of PLC with a variably reported incidence between 0.4 and 14.2%, and its overall prognosis is worse than that of either HCC or ICC alone (5, 6). Studies have revealed that in patients with PLC undergoing liver resection surgery, the survival outcome of cHCC-ICC is worse than that of HCC and that it is similar to or worse than that of ICC patients (7). HCC patients who meet the Milan criteria are indicated for liver transplantation, and their transplantation effect is excellent (8). However, increasing evidence indicates that the prognosis for cHCC-ICC patients undergoing liver transplantation is worse than that of patients with HCC alone and that cHCC-ICC is regarded as a relative contraindication for liver transplantation (9–11). Considering the scarcity of liver sources available for transplantation and the poor prognosis for cHCC-ICC, the correct identification of different PLC subtypes before surgery is a necessary condition for the reasonable selection of surgical candidates for liver transplantation and liver resection surgery, and it can improve overall survival outcomes (12, 13). PLC is often diagnosed as advanced, and many patients do not qualify for a curable treatment; systemic treatments that are effective for either HCC or ICC alone appears to be ineffective for cHCC-ICC (5). Therefore, precise and proper preoperative diagnosis is important for patient management to distinguish cHCC-ICC from HCC and ICC since different PLC subtypes may determine different treatment decisions.

Due to the high heterogeneity in the proportion and existing forms of the two tumor components, the imaging manifestations of cHCC-ICC have lacked specificity. At present, most cases of cHCC-ICC are misdiagnosed as simple HCC or ICC. Theodora et al. showed that the liver imaging reporting and data system (LI-RADS) as a common method for qualitative diagnosis of liver tumors applied in liver-contrast-enhanced ultrasound (CEUS) diagnosis may misdiagnose 54.1% of cHCC-ICC lesions as HCC (14). In contrast-enhanced imaging, cHCC-ICC has overlapping imaging modes with HCC and ICC. The main tissue in the tumor largely determines the main imaging features, making it difficult to distinguish cHCC-ICC from HCC and ICC (15). Moreover, most tumors can be diagnosed with core needle biopsy before surgery, but due to the different proportions of ICC and HCC in cHCC-ICC and sampling error, even histological biopsy may lead to preoperative diagnosis error and misdiagnosis of cHCC-ICC as HCC or ICC (16). Therefore, although accurate preoperative diagnosis of the three subtypes of PLC is important, it is still difficult.

Radiomics, a newly emerging concept in recent years, uses computers to extract a large amount of non-visual quantitative image information to realize the extraction of tumor features and model establishment, and it further excavates and analyzes image data information to assist doctors in diagnosis (17). Through the radiomics approach, the features that can be identified by human eyes and extracted by computers build a complementary relationship; in addition, radiomics combined with currently effective clinical evaluation indicators can improve the accuracy of medical diagnosis (18, 19). Tumor features vary from different tumor morphologies and biological behaviors. Radiomics as a method of deep mining high-dimensional image features can capture the characteristics of tumors more comprehensively, providing a feasible new method for identifying different tumors. Rafael et al. extracted 2D texture features and 3D texture features from T1-weighed MR images of 67 brain metastases and established a radiomics model using a random forest method. This model was helpful in distinguishing the primary tumors from brain metastases (breast cancer, lung cancer, and melanoma) (20). In the research by Yin et al., the radiomics model based on MR images can effectively identify different sacral tumors for preoperative identification of chordoma, giant cell tumor, and metastatic tumor (21).

Currently, the diagnosis of cHCC-ICC is usually based on postoperative pathology. Radiomics studies based on ultrasound evaluation of three different PLC subtypes are still lacking, and relevant reports have not been reported. In different imaging examinations, ultrasound technology has the advantages of no radiation, real-time observation, and simplicity with regard to liver disease examinations. An ultrasound-based radiomics approach may be better than other approaches in identifying three types of PLC to provide additional information. In this study, an ultrasound-based machine learning method was used to extract radiomics features and develop radiomics models to identify different pathological types of PLC.

## Materials and Methods

### Study Population

This study was approved by the Ethics Committee of the First Affiliated Hospital of Guangxi Medical University. A comprehensive retrospective research was implemented on the medical records of patients diagnosed with PLC after surgery in the First Affiliated Hospital of Guangxi Medical University from January 2017 to September 2019.

The following inclusion and exclusion criteria were implemented in this study. Inclusion criteria included the following: (1) the lesions were primary liver tumors; (2) the target nodule was confirmed by surgery pathology; (3) liver ultrasound examination was performed within 14 days before resection; and (4) the target lesions were displayed clearly on the ultrasound images. Exclusion criteria included the following: (1) anticancer treatment before surgery; (2) poor image quality; and (3) uncompleted clinical data.

**FIGURE 1:**
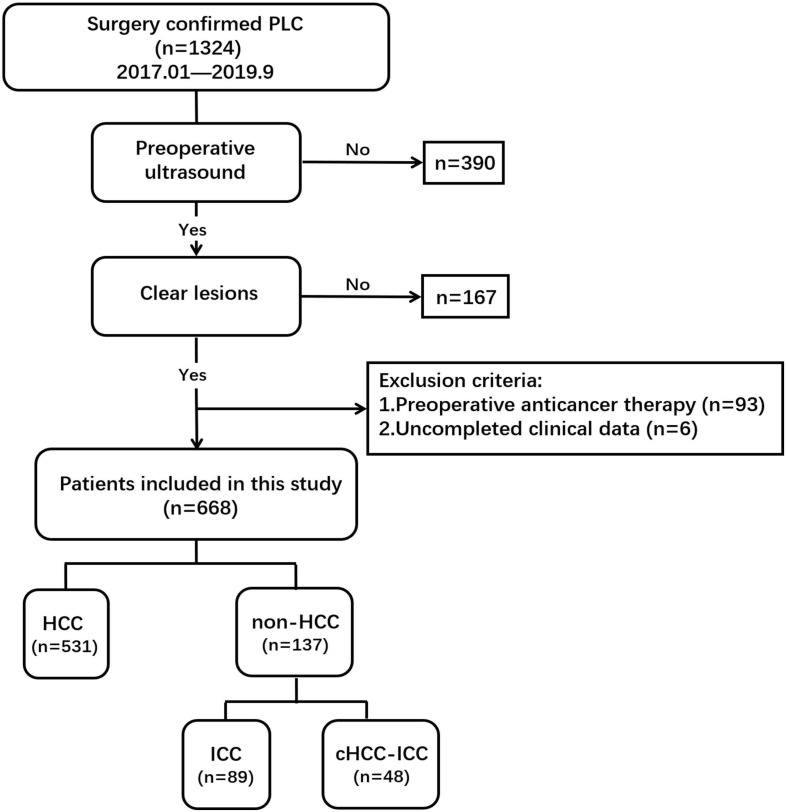
Flow chart of study population screening.

Finally, 668 eligible patients (544 male/124 female; mean age, 50.5 ± 11.4 years; age range, 22–79 years) were enrolled (Figure 1). The pathological tissue of the lesions was obtained by surgical hepatic resection for pathological diagnosis to determine the histological classification of PLC, of which there were 531 HCC patients, 89 ICC patients, and 48 cHCC-ICC patients.

### Patient Clinical Pathological Parameters

Basic patient information was collected including data on gender, age, tumor size, cirrhosis, hepatitis, and serum tumor markers. Serological data included carbohydrate antigen 19-9 (CA19-9), alpha fetoprotein (AFP), and carcinoembryonic antigen (CEA) levels. These data were measured within 2 weeks before surgery.

We also collected patient pathological information, including tumor differentiation, microvascular invasion (MVI), TNM stage, and immunohistochemical information on Ki67, p53, and vascular endothelial growth factor (VEGF). MVI referred to the observation of a nest of cancer cells in a blood vessel lining the endothelial cells by microscopy. In this study, the TNM staging of PLC patients was analyzed according to the American Joint Cancer Commission (AJCC) eighth edition staging system (22, 23).

### Radiomics Analysis

**FIGURE 2:**
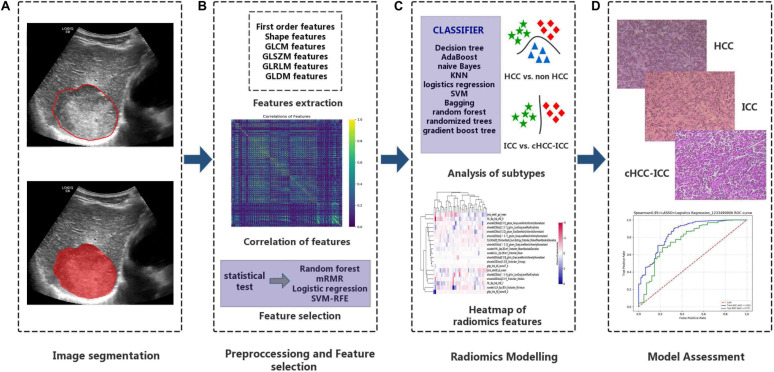
Important steps for the radiomics research. **(A)** Tumor regions-of-interest identification. **(B)** After the tumor images are digitalized, a total of 5,234 quantitative features were obtained, and data were standardized for preprocessing. **(C)** The combination of dimension reduction and classifier was performed to develop radiomics models to identify primary liver cancers of different histopathological types. **(D)** Evaluation of the classification effects of the radiomics model in identifying different histopathological types of primary liver cancer.

The research of radiomics mainly includes the following steps: tumor segmentation, data preprocessing and feature selection, modeling, and evaluation (Figure 2). In the training cohort, we performed a combination of different dimension-reduction technologies and machine learning approaches to establish radiomics models. Finally, the test cohort was taken to evaluate the generalization performance of the model.

### Ultrasound Imaging and Tumor Segmentation

**FIGURE 3:**
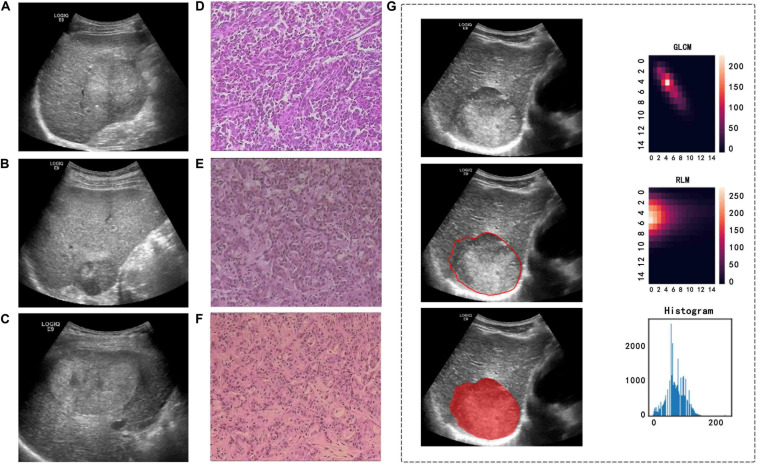
Ultrasound and pathological images, tumor segmentation, and feature extraction of three different pathological types of PLC. **(A,D)** A 57-year-old man with a pathological diagnosis of cHCC-ICC. **(B,E)** A 64-year-old woman with a pathological diagnosis of HCC. **(C,F)** A 44-year-old man with a pathological diagnosis of ICC. **(G)** An example of manually sketching the region of interest (ROI) of a tumor on an ultrasound image and gray level co-occurrence matrix (GLCM) features, run length matrix (RLM) features, and grayscale histogram feature extraction from the grayscale ultrasound image.

GE Logiq E9 ultrasound diagnostic instruments (GE Healthcare, United States, C5-1 abdominal probe, 2.8–5.0 MHz), Philips EPIQ 5 ultrasound diagnostic instruments (Philips Medical Systems, United States, C5-1 abdominal probe, 1–5 MHz), and Aloka EZU-MT28-S1 ultrasound diagnostic instruments (Aloka, Japan, abdominal probe, 2–6 MHz) were used to collect images. We conducted a retrospective review of the image data and selected two-dimensional ultrasound images in digital imaging and communications in medicine (DICOM) format that clearly showed the largest cross section of each lesion. We imported the images into the ITK-SNAP software (version 3.8.0)^1^ to manually draw the tumor boundary and determine the tumor region of interest (ROI) (Figure 3). Under the supervision of a radiologist with over 20 years of ultrasound diagnosis experience, another radiologist with 15 years of ultrasound diagnosis experience completed the ROIs for all tumors.

### Feature Extraction and Data Preprocessing

Intelligence Foundry software (GE Healthcare, version 1.3) was used for radiomics analysis. Since the images were collected by different ultrasound equipment and the feature vectors had a wide range, we preprocessed the data before modeling analysis to improve the accuracy of the calculation, including ultrasound system supplier data alignment, median value replacement of missing value processing, and data normalization processing.

We used 256 as the bin size to discretize the gray value of the images and used the ComBat method to standardize the radiomics features. The ComBat method was previously used in radiomics studies of different PTE or MRI protocols (24, 25). The wavelet features were based on the original gray value image for wavelet transformation (including HLH, LLL, and HHL, with eight local matrices); the energy, skewness, and other series of parameters were extracted from the obtained wavelet transform matrix. In the same way, the shearlet change and the gabor operator transformation were also carried out, and different step lengths were used in the change to obtain multiple sets of transformation intermediate value matrices. Based on the above transformations, the radiomics parameters were extracted, and finally, we obtained 5,234 high-throughput features. The types of features included the following: first-order features (energy, mean, skewness, kurtosis, etc.), shape features (minor axis length, major axis length, elongation, etc.), wavelet features and textural features [gray level co-occurrence matrix (GLCM) features, grey level run length matrix (GLRLM) features, etc] (Supplementary Part A). The feature parameters extracted by the Intelligence Foundry software (GE Healthcare, version 1.3) were algorithms provided using the pyradiomics package, which calculated the radiomics features in accordance with the feature definition described in the 2016 version of the image biomarker standardization initiative (IBSI) (26, 27). The median was used to fill in missing extracted feature values and substitute abnormal value. *Z*-score normalization was used to convert different data to the same order of magnitude, and the calculation formula was as follows:

y=(x-μ)/σ

where *μ* is the mean and *σ* is the standard deviation.

The PLC patients were labeled according to different histological types into different layers. In the HCC-vs-non-HCC model, the non-HCC label was “0,” and the HCC label was “1.” In the ICC-vs-cHCC-ICC model, the cHCC-ICC label was “0,” and the ICC label was “1.” Then, PLC patients with different histological types were grouped based on a 7:3 ratio (training cohort : test cohort) in each layer using the method of stratified sampling. The training cohort was used to build the model, and the test cohort was an independent external verification to evaluate the model established by the training cohort.

### Feature Selection

We obtained 5,234 high-throughput radiomics features and normalized the quantitative expression values of the radiomics features using the *Z*-score method. Considering that some highly correlated and redundant features in the data may affect the classification effect of the model, we calculated the Spearman correlation coefficient. A correlation coefficient between the two variables close to 1 indicated that the linear relationship between them was strong and that one of the two variables could be used instead of the other. In this study, the high-correlation features were removed with a threshold of 0.95 (HCC vs. non-HCC) and 0.75 (ICC vs. cHCC-ICC). Then, we used the statistical test method to screen for features that had differences.

Finally, we used four dimension-reduction technologies to further deal with the features that were processed above. Dimension-reduction technologies included random forest, max-relevance and min-redundancy (mRMR), logistic regression, and support vector machine recursive feature elimination (SVM-RFE) (Supplementary Part B).

### Modeling and Evaluation

The final selected radiomics features were imported into the classifier to build a model for evaluating three different histopathological types of PLC. Ten machine learning approaches were used in this study, which were decision tree, naïve Bayes, k-nearest neighbor (KNN), logistics regression, support vector machine (SVM), bagging, random forest, extremely randomized trees, AdaBoost, and gradient boosting tree (Supplementary Part B).

We extracted 5,234 features from the ultrasound images. We quantify the discriminative ability of the radiomics model by calculating the receiver operating characteristic curve (AUC). We constructed the model by separately combining the above four dimension-reduction technologies and the above 10 machine learning approaches and chose the combination with the highest AUC to build the optimal radiomics model. In the training cohort, to avoid overfitting the classifier, we used a 10-fold cross-validation method.

We performed a receiver operating characteristic (ROC) curve analysis and calculated the accuracy and precision. We also used the confusion matrix as a measure of the quality of the machine learning approaches to verify whether the prediction results were consistent with the actual results. The confusion matrix is a useful tool for evaluating the classification ability of radiomics models (28).

In the HCC-vs-non-HCC and ICC-vs-cHCC-ICC radiomics models, we performed univariate and multivariate logistic regression analyses to analyze the relevant factors of different pathological types of PLC. Univariate analysis factors with *P*-values less than 0.1 were further analyzed by multivariate logistic regression analysis. In multivariate analysis, a *P*-value less than 0.05 was considered significant.

### Statistical Analysis

R software (version 3.6.0) and SPSS software (version 22.0) were applied for statistical analysis. In the quantitative data with a normal distribution, the completely random design *t*-test was performed for the two-samples contrast, the analysis of variance was used to contrast several independent samples, and variables were summarized as the mean ± standard deviation (SD). For quantitative data with a skewed distribution, the Mann–Whitney *U* test was performed to compare two independent samples, the Kruskal–Wallis *H* test was used to compare several independent samples, and variables were summarized as the median (q1–q3). Qualitative data were compared using chi-square tests, with variables described as percentages. *P*-values below 0.05 was considered to be statistically significant differences. In the R software (version 3.6.3), the “pheatmap” package was used to draw heat maps of features.

## Results

### Clinicopathological Data of PLC Patients

A total of 668 PLC patients were adopted in this research (Figure 1). The clinicopathological parameters of the training and test cohorts were shown in Table 1. There were no significant differences in the distribution of clinicopathological features between the two cohorts, including gender, age, tumor size, hepatitis, cirrhosis, serum tumor markers, pathological subtype, immunohistochemistry, or tumor stage. These results showed the rationality of our training and test cohort partitions.

**TABLE 1:** Clinicopathological profiles of two radiomics models in the training cohort and test cohort.

	HCC vs. non-HCC Model				ICC vs. cHCC-ICC Model		
			
Variables	Training cohort (*n* = 467)	Test cohort (*n* = 201)	*P*-value	Variables	Training cohort (*n* = 95)	Test cohort (*n* = 42)	*P*-value
**Gender**				**Gender**			

Male	379 (81.2)	165 (41.8)	0.78	Male	65 (68.4)	27 (64.3)	0.63
Female	88 (18.8)	36 (58.2)		Female	30 (31.6)	15 (35.7)	

**Age (years)**				**Age (years)**			

<40	88 (18.8)	35 (174)	0.89	<40	21 (22.1)	5 (11.9)	0.27
40–60	280 (60.0)	124 (61.7)		40–60	59 (62.1)	27 (64.3)	
>60	99 (21.2)	42 (20.9)		>60	15 (15.8)	10 (23.8)	

**Tumor size (cm)**				**Tumor size (cm)**			

≤5	246 (52.7)	115 (57.2)	0.28	≤5	40 (42.1)	15 (35.7)	0.48
>5	221 (47.3)	86 (42.8)		>5	55 (57.9)	27 (64.3)	

**Hepatitis**				**Hepatitis**			

Yes	364 (80.2)	159 (79.9)	0.15	Yes	58 (61.1)	23 (54.8)	0.09
No	103 (19.8)	59 (20.1)		No	37 (38.9)	19 (45.2)	

**Cirrhosis**				**Cirrhosis**			

Yes	218 (46.7)	108 (53.7)	0.09	Yes	36 (37.9)	14 (33.3)	0.61
No	249 (53.3)	93 (46.3)		No	59 (62.1)	28 (66.7)	

**AFP (μ g/ml)**				**AFP (μ g/ml)**			

≤400	344 (73.7)	139 (69.2)	0.23	≤400	76 (80.0)	38 (90.5)	0.13
>400	123 (26.3)	62 (30.8)		>400	19 (20.0)	4 (9.5)	

**CA19-9 (U/ml)**				**CA19-9 (U/ml)**			

≤37	389 (83.3)	158 (78.6)	0.15	≤37	62 (65.3)	23 (54.8)	0.24
>37	78 (16.7)	43 (21.4)		>37	33 (34.7)	19 (45.2)	

**CEA (μ g/ml)**				**CEA (μ g/ml)**			

≤5	411 (88.0)	179 (89.1)	0.70	≤5	75 (78.9)	33 (78.6)	0.96
>5	56 (12.0)	22 (10.9)		>5	20 (21.1)	9 (21.4)	

**Histological type**				**Histological type**			

HCC	371 (79.4)	160 (79.6)	0.96	cHCC-ICC	33 (34.7)	15 (35.7)	0.91
Non-HCC	96 (20.6)	41 (20.4)		ICC	62 (65.3)	27 (64.3)	

**Differentiation**				**Differentiation**			

Well	21 (4.5)	5 (2.5)	0.61	Well	1 (1.0)	0 (0)	0.87
Moderate	331 (70.9)	149 (74.1)		Moderate	60 (63.2)	25 (59.5)	
Poor	83 (17.8)	33 (16.4)		Poor	23 (24.2)	11 (26.2)	
No data	32 (6.8)	14 (7.0)		No data	11 (11.6)	6 (14.3)	

**Immunohistochemistry, Positive/Negative**				**Immunohistochemistry, Positive/Negative**			

Ki67, >10%/≤10%	286/181 (61.2/38.8)	129/72 (64.2/35.8)	0.47	Ki67, >10%/≤10%	46/14 (48.4/51.6)	32/10 (76.2/23.8)	0.96
P53	254/213 (54.4/45.6)	122/79 (60.7/39.3)	0.13	P53	63/32 (66.3/33.7)	28/14 (66.7/33.3)	0.96
VEGF	219/248 (46.9/53.1)	102/99 (50.7/49.3)	0.36	VEGF	40/55 (42.1/57.9)	20/22 (47.6/52.3)	0.55
Microvascular invasion	132/335 (28.3/71.7)	62/139 (30.8/69.2)	0.50	Microvascular invasion	33/62 (34.7/65.3)	11/31 (26.2/73.8)	0.32
**Depth of invasion**				**Depth of invasion**			

T1	255 (54.6)	111 (55.2)	0.53	T1	35 (36.8)	24 (57.2)	0.15
T2	111 (23.8)	55 (27.4)		T2	30 (31.6)	8 (19.0)	
T3	6 (1.3)	3 (1.5)		T3	1 (1.1)	0 (0)	
T4	95 (20.3)	32 (15.9)		T4	29 (30.5)	10 (23.8)	

**Lymph node metastasis**				**Lymph node metastasis**			

N0	450 (96.4)	194 (96.5)	0.92	N0	81 (85.3)	38 (90.5)	0.40
N1	17 (3.6)	7 (3.5)		N1	14 (14.7)	4 (9.5)	

**Distant metastasis**				**Distant metastasis**			

M0	451 (96.6)	198 (98.5)	0.17	M0	87 (91.6)	40 (95.2)	0.45
M1	16 (3.4)	3 (1.5)		M1	8 (8.4)	2 (4.8)	

**Stage**				**Stage**			

I	251 (53.7)	107 (53.2)	0.38	I	30 (31.6)	23 (54.8)	0.08
II	107 (22.9)	54 (26.9)		II	27 (28.4)	7 (16.7)	
III	89 (19.1)	36 (17.9)		III	30 (31.6)	10 (23.8)	
IV	20 (4.3)	4 (2.0)		IV	8 (8.4)	2 (4.7)	

**Radiomics score**	1.58 (0.97–2.04)	1.58 (0.95–2.10)	0.74	**Radiomics score**	0.91 (−0.31−2.39)	0.85 (−0.40−1.76)	0.29

*Values were shown as the number of patients (percentage) unless otherwise explained. Radiomics score data were shown as median (Q1 – Q3). AFP, alpha fetoprotein; CA19-9, carbohydrate antigen 19-9; CEA, carcino-embryonic antigen; VEGF, vascular endothelial growth factor.*

In the HCC-vs-non-HCC group, the study sample included 467 people in the training cohort (379 male/88 female, mean age, 50.5 ± 11.4 years), 371 cases of HCC, and 96 cases of non-HCC. There were 201 patients in the test cohort (165 male/36 female, mean age, 50.6 ± 11.3 years), 160 cases of HCC, and 41 cases of non-HCC. In the ICC-vs-cHCC-ICC group, the study sample included 95 people in the training cohort (65 male/30 female, mean age, 49.4 ± 11.6 years), 33 cases of cHCC-ICC and 62 cases of ICC. There were 42 patients in the test cohort (27 male/15 female, mean age, 51.8 ± 10.3 years), 15 cases of cHCC-ICC, and 27 cases of ICC.

### Identification of the Radiomics Signature

**FIGURE 4:**
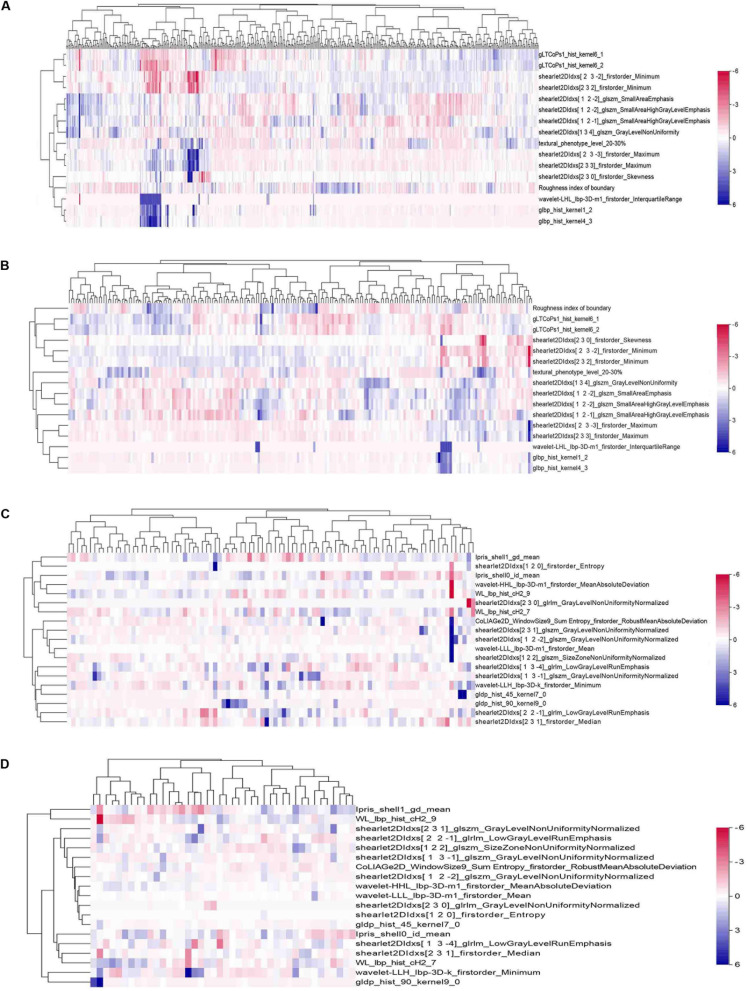
Heat maps of the final features of radiomics models. A total of 16 features were used to build the HCC-vs-non-HCC model, and 19 features were used to build the ICC-vs-cHCC-ICC model. The radiomics features were normalized by *Z*-score. **(A)** Training cohort in the HCC-vs-non-HCC model. **(B)** Test cohort in the HCC-vs-non-HCC model. **(C)** Training cohort in the ICC-vs-cHCC-ICC model. **(D)** Test cohort in the ICC-vs-cHCC-ICC model.

In the HCC-vs-non-HCC group, we used the LASSO regression method for dimension reduction and modeling with the logistics regression method. In the ICC-vs-cHCC-ICC group, we used the random forest method for dimension reduction, feature selection with a threshold value of 1.25 times the mean value, and modeling with the logistics regression method. Finally, we respectively identified 16 and 19 optimal radiomics features for HCC-vs-non-HCC model and ICC-vs-cHCC-ICC model predictions (Table 2). Figure 4 showed the heat map of 16 features (HCC-vs-non-HCC model) and 19 features (ICC-vs-cHCC-ICC model) of the final radiomics models.

**TABLE 2:** Features and corresponding coefficients of HCC vs. non-HCC radiomics model and ICC vs. cHCC-ICC radiomics model.

HCC vs. non-HCC Model		ICC vs. cHCC-ICC Model	
	
Radiomics features	Coefficient	Radiomics features	Coefficient
Roughness index of boundary	–0.034	Ipris_shell0_id_mean	0.019
Textural_phenotype_level_20–30%	–0.423	Ipris_shell1_gd_mean	0.026
Wavelet-LHL_lbp-3D-m1_firstorder_InterquartileRange	–0.068	CoLIAGe2D_WindowSize9_Sum Entropy_firstorder_RobustMean Absolute Deviation	0.021
Shearlet2didxs[1 2 -2]_glszm_Small Area Emphasis	–0.020	Wavelet-LLH_lbp-3D-k_firstorder_Minimum	0.019
Shearlet2didxs[1 2 -2]_glszm_Small Area High GrayLevel Emphasis	–0.006	Wavelet-HHL_lbp-3D-m1_firstorder_MeanAbsoluteDeviation	0.026
Shearlet2didxs[1 2 -1]_glszm_Small Area High GrayLevel Emphasis	–0.053	Wavelet-LLL_lbp-3D-m1_firstorder_Mean	0.029
shearlet2DIdxs[1 3 4]_glszm_GrayLevel Non-Uniformity	–0.058	Shearlet2didxs[1 2 -2]_glszm_GrayLevel Non-Uniformity Normalized	0.028
Shearlet2didxs[2 3 -3]_firstorder_Maximum	–0.028	Shearlet2didxs[1 2 0]_firstorder_Entropy	0.023
Shearlet2didxs[2 3 -2]_firstorder_Minimum	0.017	Shearlet2didxs[1 2 2]_glszm_Size Zone NonUniformity Normalized	0.028
Shearlet2didxs[2 3 0]_firstorder_Skewness	–0.038	shearlet2DIdxs[1 3 −4] _glrlm_Low GrayLevel Run Emphasis	0.021
Shearlet2didxs[2 3 2]_firstorder_Minimum	0.023	Shearlet2didxs[1 3 −1]_glszm_GrayLevel Non-Uniformity Normalized	0.025
Shearlet2didxs[2 3 3]_firstorder_Maximum	–0.165	Shearlet2didxs[2 2 −1]_glrlm_Low GrayLevel Run Emphasis	0.024
glbp_hist_kernel1_2	–0.323	Shearlet2didxs[2 3 0]_glrlm_GrayLevel Non-Uniformity Normalized	0.023
glbp_hist_kernel4_3	–0.008	Shearlet2didxs[2 3 1]_firstorder_Median	0.022
gLTCoPs1_hist_kernel6_1	0.056	Shearlet2didxs[2 3 1]_glszm_GrayLevel Non-Uniformity Normalized	0.022
gLTCoPs1_hist_kernel6_2	0.061	gldp_hist_45_kernel7_0	0.019
		gldp_hist_90_kernel9_0	0.031
		WL_lbp_hist_cH2_7	0.022
		WL_lbp_hist_cH2_9	0.021

### Radiomics Model Assessment

**FIGURE 5:**
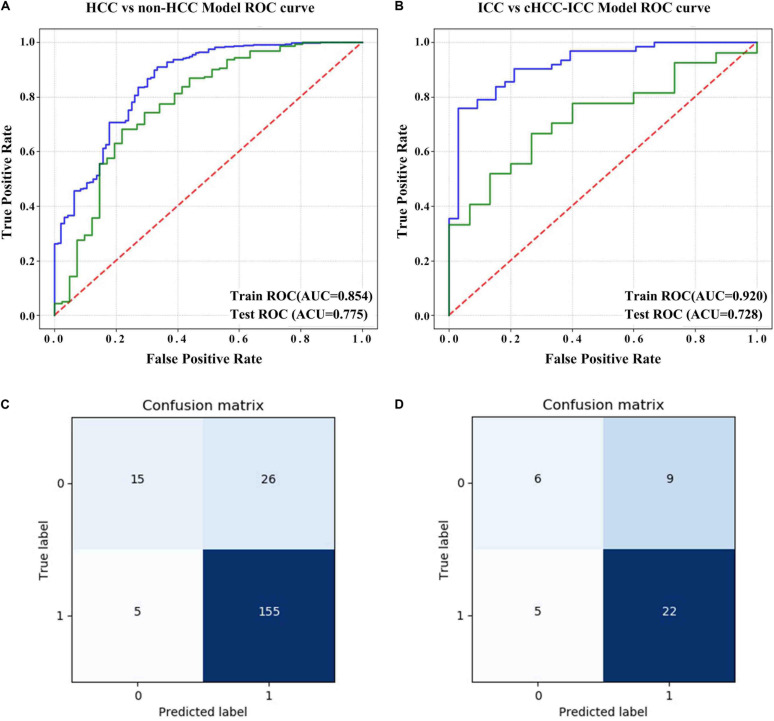
Evaluation of the predictive performance of the radiomics models. **(A)** ROC curve of the HCC-vs-non-HCC model in the training cohort and test cohort. **(B)** ROC curve of the ICC-vs-cHCC-ICC model in the training cohort and test cohort. **(C)** Confusion matrix of the HCC-vs-non-HCC model in the test cohort. The non-HCC label was “0,” and the HCC label was “1.” **(D)** Confusion matrix of the ICC-vs-cHCC-ICC model in the test cohort. The cHCC-ICC label was “0,” and the ICC label was “1.” The abscissa represents the predicted label, and the ordinate represents the actual label.

The results showed that the radiomics models we built had a high overall classification performance for identifying three subtypes of PLC. The AUC values in the training cohort and test cohort were 0.854 and 0.775 (HCC vs. non-HCC) and 0.920 and 0.728 (ICC vs. cHCC-ICC), respectively (Figures 5A,B). The confusion matrix was shown in Figures 5C,D. In the HCC-vs-non-HCC model, the predicted results showed that of the 160 actual HCC patients, 155 were correctly predicted to be HCC. In the ICC-vs-cHCC-ICC model, the 15 patients with actual cHCC-ICC, 6 were predicted to be cHCC-ICC, and among the 27 actual ICC patients, 22 were correctly predicted to be ICC. These results indicated that the radiomics models can moderately distinguish three different histological types of PLC and performed best at HCC identification.

Tables 3, 4 showed the results of univariate and multivariate logistic regression analyses of HCC-vs-non-HCC and ICC-vs-cHCC-ICC radiomics models. In the HCC-vs-non-HCC radiomics model, gender, hepatitis, AFP, CA19-9, CEA, stage, and radiomics score were independent factors related to HCC (*P* < 0.05). In the ICC-vs-cHCC-ICC radiomics model, AFP and radiomics score were independent factors related to cHCC-ICC (*P* < 0.05).

**TABLE 3:** Results of the univariate and multivariate analyses in HCC-vs-non-HCC Model.

Factors (reference)	Univariate analysis		Multivariate analysis	
		
	OR (95% CI)	*p* value	OR (95% CI)	*p* value
**Gender (female)**	0.357 (0.233–0.549)	0.000*	0.379 (0.190–0.758)	0.006
**Age (years)**				
<40	Reference		−	−
40–60	0.804 (0.436–1.482)	0.485	−	−
>60	0.797 (0.487–1.305)	0.367	−	−
**Tumor size (>5 cm)**	0.508 (0.347–0.745)	0.001	1.618 (0.855–3.061)	0.139
**Hepatitis (yes)**	3.433 (2.279–5.172)	0.000*	2.642 (1.360–5.133)	0.004
**Cirrhosis (yes)**	1.883 (1.279–2.774)	0.001	1.436 (0.775–2.661)	0.250
**AFP (μg/ml)**				
≤400	Reference		Reference	
>400	2.176 (1.340–3.533)	0.002	3.533 (1.702–7.335)	0.001
**CA19-9 (U/ml)**				
≤37	Reference		Reference	
>37	0.244 (0.159–0.374)	0.000*	0.232 (0.118–0.456)	0.000*
**CEA (μg/ml)**				
≤5	Reference		Reference	
>5	0.379 (0.229–0.627)	0.000*	0.427 (0.189–0.965)	0.041
**Differentiation**				
Well	Reference		Reference	
Moderate	10.366 (1.350–79.590)	0.025	4.266 (0.361–50.333)	0.249
Poor	1.927 (1.212–3.063)	0.006	1.681 (0.867–3.258)	0.124
**Immunohistochemistry, Negative/Positive**				
Ki67, ≤10%/>10%	0.407 (0.263–0.629)	0.000*	0.632 (0.306–1.303)	0.214
P53	0.586 (0.395–0.868)	0.008	0.531 (0.275–1.025)	0.059
VEGF	1.241 (0.850–1.810)	0.264	−	−
Microvascular invasion	0.832 (0.555–1.248)	0.374	−	−
**Stage**				
I	Reference		Reference	
II	4.111 (1.735–9.736)	0.001	4.077 (1.152–14.425)	0.029
III	2.668 (1.090–6.532)	0.032	5.245 (1.410–19.504)	0.013
IV	1.518 (0.621–3.712)	0.360	2.267 (0.616–8.342)	0.218
**Radiomics score**	3.555 (2.789–4.532)	0.000*	4.295 (3.098–5.953)	0.000*

In univariate analysis, variables with *P* < 0.1 were included in multivariate logistic regression analysis. In multivariate analysis, *P* < 0.05 was considered significant. AFP, alpha fetoprotein; CA19-9, carbohydrate antigen 19-9; CEA, carcino-embryonic antigen; VEGF, vascular endothelial growth factor. * represents *P* < 0.0001.

**TABLE 4:** Results of the univariate and multivariate analyses in ICC-vs-cHCC-ICC Model.

Factors (reference)	Univariate analysis		Multivariate analysis	
		
	OR (95% CI)	*p* value	OR (95% CI)	*p* value
**Gender (female)**	2.943 (1.272–6.814)	0.012	1.924 (0.638–5.806)	0.245
**Age (years)**				
<40	Reference		−	−
40–60	0.431 (0.129–1.434)	0.170	−	−
>60	0.560 (0.202–1.551)	0.265	−	−
**Tumor size (>5 cm)**	1.781 (0.874–3.632)	0.112	−	−
**Hepatitis (yes)**	0.246 (0.109–0.554)	0.001	0.572 (0.178–1.832)	0.347
**Cirrhosis (yes)**	0.413 (0.199–0.854)	0.017	0.700 (0.232–2.112)	0.527
**AFP (μg/ml)**				
≤400	Reference		Reference	
>400	0.198 (0.077–0.507)	0.001	0.205 (0.057–0.735)	0.015
**CA19-9 (U/ml)**				
≤37	Reference		Reference	
>37	2.449 (1.128–5.318)	0.024	1.222 (0.400–3.740)	0.725
**CEA (μg/ml)**				
≤5	Reference		Reference	
>5	6.190 (1.765–21.711)	0.004	4.554 (0.919–22.571)	0.063
**Differentiation**				
Well/Moderate	Reference		−	−
Poor	2.174 (0.849–5.569)	0.106	−	−
**Immunohistochemistry, Negative/Positive**				
Ki67, ≤ 10%/> 10%	0.703 (0.294–1.678)	0.427	−	−
P53	0.733 (0.344–1.565)	0.423	−	−
VEGF	0.523 (0.257–1.065)	0.074	0.570 (0.211–1.540)	0.267
Microvascular invasion	0.596 (0.284–1.250)	0.171	−	−
**Stage**				
I	Reference		−	−
II	0.302 (0.059–1.559)	0.153	−	−
III	0.281 (0.052–1.523)	0.141	−	−
IV	1.417 (0.240–8.367)	0.701	−	−
**Radiomics score**	2.292 (1.662–3.160)	0.000*	2.395 (1.636–3.506)	0.000*

In univariate analysis, variables with *P* < 0.1 were included in multivariate logistic regression analysis. In multivariate analysis, *P* < 0.05 was considered significant. AFP, alpha fetoprotein; CA19-9, carbohydrate antigen 19-9; CEA, carcino-embryonic antigen; VEGF, vascular endothelial growth factor. * represents *P* < 0.0001.

## Discussion

In this research, as far as we know, we are the first to identify an ultrasound-based radiomics models that can be used to predict HCC, ICC, and cHCC-ICC. The radiomics models achieved good diagnostic efficiency in both the training cohort and the test cohort, which is expected to help doctors improve the accuracy of presurgical diagnosis and guide the further treatment of PLC patients.

Another highlight of this study is that we constructed the optimal model through a variety of combinations of dimension-reduction technologies and classifiers. Shiri et al. found that the performance of machine learning models depends on the type of data or application and that there was no general algorithm or single model (29). Different combinations of feature selection methods and classifiers can provide different results (30–32). In the current study, we performed different dimension-reducing technologies and machine learning approaches to find the optimal models to predict HCC vs. non-HCC and ICC vs. cHCC-ICC. Therefore, the models that we obtained comprehensively captured the potential of radiomics-based differential diagnosis of PLC in the current clinical medical environment.

In the current clinical practice, physicians preoperatively rely on clinical symptoms, tumor serum markers, and imaging tests to determine the type of PLC patient, but these data can sometimes lead to false diagnoses because they may overlap. In addition, due to high heterogeneity in the proportion and existing forms of the two tumor components, the imaging findings of mixed HCC currently lack performance, and most cases are misdiagnosed as simple HCC or ICC. Preoperative differentiation of PLC subtypes has important clinical significance, as different types are associated with different treatment options and prognosis. Improving the accuracy of initial diagnosis can provide more optimized and active treatment for cHCC-ICC patients (16). In addition, clinical medicine is currently moving toward a trend of precision and personalized medicine. In the precise medical environment, medical imaging as an important diagnostic tool is also rapidly evolving and gradually playing an important role (33). Radiomics, which provides a non-invasive method to assess lesions and performs well in the diagnosis and prediction of tumors, is widely considered to be a step in the evolution of imaging toward a concept of personalized cancer management (34, 35).

So far, only a few studies have attempted to identify three different tissue types of PLC by imaging methods, and most previous studies have been based on CT and MR images. Wang et al. previously attempted to use preoperative CT and MR imaging to identify cHCC-ICC with HCC and ICC. The study found that compared with ICC and cHCC-ICC, the incidence of HCC pseudocapsule was significantly higher. Compared with their occurrence in HCC and cHCC-ICC, rim enhancement, abnormal perfusion, capsular retraction, and biliary dilatation were more common in ICC. However, in that study, the number of features obtained from images was small, and imaging features, such as tumor size, were all visible to the naked eye; the approach failed to identify and analyze microscopic image features with potential value for clinical diagnosis (36). Lewis et al. used MR images of 65 liver cancer patients. The tumor characteristics and LI-RADS classification were evaluated by two independent observers. Among the two independent observers, the combined AUC of sex and LI-RADS and apparent diffusion coefficient (ADC) at the fifth percentile for the diagnosis of liver cancer were 0.90 and 0.89, respectively. This result showed that HCC can be better distinguished from ICC and cHCC-ICC by combining the ADC histogram parameters and LI-RADS categorization. However, the number of samples included in that study and the number of extracted features were small, and the study did not distinguish between ICC and cHCC-ICC (37). Compared with CT/MRI, ultrasound examination has the advantages of simplicity and real-time observation, and it plays a vital role in the diagnosis and treatment of liver tumors. However, no radiomics study has sought to identify HCC, cHCC-ICC, and ICC. In view of this knowledge gap, we used ultrasound images to establish radiomics models to distinguish three different pathological classifications of PLC, and we obtained promising results.

Our results showed that the radiomics models we built have a good overall AUC and could well to accurately predict pure HCC, while obtaining lower accuracy in cHCC-ICC. Our findings are roughly consistent with the results of some previous studies that suggest that identifying cHCC-ICC from PLC remains challenging, possibly due to the greater histological heterogeneity of cHCC-ICC. Wang et al. studied the CT and MR images of 136 patients with PLC and found that the features of capsular retraction, abnormal perfusion, and rim enhancement showed better performance in the identification of HCC and ICC, while the ability to distinguish cHCC-ICC from the other two types of PLC was not significant (36). Many image features such as shape, size, edge, position, and enhancement mode in cHCC-ICC mostly behave like ICC or HCC, creating some difficulties in its diagnosis (38).

We finally used the LASSO and random forest methods for feature selection. LASSO regression is also called L1 regularization of linear regression, which is a popular method used in radiomics researches. The basic idea of LASSO is to minimize the residual sum of squares under the constraint that the sum of the absolute values of the regression coefficients is less than a constant, so as to produce some regression coefficients strictly equal to 0 to get an interpretable model. Essentially, it is a process of seeking a sparse expression of the model (39, 40). Random forest is an ensemble learning algorithm based on decision tree analysis and has a good performance in classification and regression. Random forest can also be used as a feature selection technology, and it has been widely used in machine learning, determining the importance of features during model training (28, 41, 42).

The texture features showed high importance in our prediction model. Image texture is a visual feature that reflects homogeneous phenomena in the image, and it reflects the surface structure organization and arrangement properties of the object with slow or periodic changes. The texture can be layered by the statistical order of the information encoded in the image, which can be divided into first-order texture features, second-order texture features, and high-order texture features (43). Texture features are widely recognized as quantitative biomarkers of tumor heterogeneity (44, 45).

The large sample size of our study helped to improve the generality and stability of our results. However, our research also has certain limitations. First, all ultrasound imaging data were from a unitary center, and the study was retrospective in nature. The grayscale ultrasound images used in our study were collected by different commercial ultrasound systems. Although the data extracted from the images were preprocessed, the imaging of different instruments may still have some influence on the results of feature extraction, so whether the model can play a prospective role remains an open question. Therefore, it is necessary to conduct a multicenter prospective study with a rigorous control of ultrasound machines to further explore the diagnostic potential of radiomics-based modeling. Second, our study included only PLC and did not include benign and metastatic tumors of the liver. The identification of more types of tumors is more challenging. We will add data for other types of liver tumors in future studies to optimize the universality and clinical value of the model. Third, we took into account the characteristics of general clinical applications of ultrasound, and this is a retrospective study, so we finally adopted two-dimensional ultrasound images. However, the quantitative features extracted based on two-dimensional ultrasound images cannot stand for the overall lesion, and a more precise radiomics analysis depends on the acquisition of 3D images. Further research on three-dimensional ultrasound radiomics is necessary in the future. Fourth, our study focused on the relationship between high-throughput imaging features extracted from tumor ROI and pathological typing. In order to quantify the heterogeneity of tumors more comprehensively, it is necessary to pay more attention to the peritumoral information and combine more clinicopathological information to establish a more accurate individualized disease assessment model. Therefore, in the future, we need to optimize our model based on the above limitations and carry out prospective studies, which may be helpful to improve the discrimination performance of radiomics model for PLC.

In summary, we developed and validated the ultrasound-based radiomics models to distinguish different histopathological types of PLC, thus providing a new approach for doctors to non-invasively identify HCC, cHCC-ICC, and ICC.

## Data Availability Statement

The raw data supporting the conclusion of this manuscript will be made available by the authors, without undue reservation, to any qualified researcher.

## Ethics Statement

This study was approved by the Ethics Committee of the First Affiliated Hospital of Guangxi Medical University.

## Author Contributions

HY and YH: guarantor of the article. HY, YH, YP, and PL: conception and design. YP, PL, LW, YZ, LL, XM, DW, YL, and HQ: collection and assembly of data. YP, PL, LW, XL, and XW: data analysis and interpretation. All authors: manuscript writing and final approval of manuscript.

## Conflict of Interest

XL and XW were employed by GE Healthcare. The remaining authors declare that the research was conducted in the absence of any commercial or financial relationships that could be construed as a potential conflict of interest. The handling editor declared a past co-authorship with one of the authors XL.

## Footnotes

1 http://www.itksnap.org
