# Supplementary material for: Ultrasound-Based Radiomics Analysis for Preoperatively Predicting Different Histopathological Subtypes of Primary Liver Cancer
Source: Front Oncol. 2020 Sep 24;10:1646. doi: 10.3389/fonc.2020.01646 (PMC7543652; doi:10.3389/fonc.2020.01646)
Supplement: Supplementary file 1 [file Presentation_1.pdf]

Part A. Radiomics Features

1．First-order features

Energy, Total energy, Entropy, Minimum, 10th percentile, 90th percentile, Maximum, Mean, Median, Interquartile range, Range, Mean absolute deviation (MAD), Robust mean absolute deviation(rMAD), Root mean squared (RMS), Standard deviation, Skewness, Kurtosis, Variance, Uniformity.

2．Shape features

Volume, Surface area, Surface area to volume ratio, Sphericity, Compactness 1, Compactness 2, Spherical disproportion, Maximum 2D diameter (slice), Maximum 2D diameter (column), Maximum 2D diameter (row), Major axis, Minor axis, Least axis, Elongation, Flatness.

3．Wavelet features and Textural features

Gray-level co-occurrence matrix (GLCM):

Autocorrelation, Joint average, Cluster prominence, Cluster shade, Cluster tendency, Contrast, Correlation, Difference average, Difference entropy, Difference variance, Joint energy, Joint entropy, Informal measure of correlation (IMC) 1, Informal measure of correlation (IMC) 2, Inverse difference moment (IDM), Inverse Difference Moment Normalized (IDMN), Inverse difference (ID), Inverse Difference Normalized (IDN), Inverse variance, Maximum probability, Sum average, Sum entropy, Sum of squares.

Gray-level run length matrix (GLRLM):

Short-run emphasis (SRE), Long-run emphasis (LRE), Gray-Level Non-Uniformity (GLN), Gray-Level Non-Uniformity Normalized (GLNN), Run Length Non-Uniformity (RLN), Run Length Non-Uniformity Normalized (RLNN), Run percentage (RP), Gray-level variance (GLV), Run variance (RV), Run entropy (RE), Low Gray-Level Run Emphasis (LGLRE), High Gray-Level Run Emphasis (HGLRE), Short-Run Low Gray-Level Emphasis (SRLGLE), Short-Run High Gray-Level Emphasis (SRHGLE),Long-Run Low Gray-Level Emphasis (LRLGLE), Long-Run High Gray-Level Emphasis (LRHGLE).

Gray-Level Size Zone Matrix (GLSZM):

Small Area Emphasis (SAE), Large Area Emphasis (LAE), Gray-Level Non-Uniformity (GLN), Gray-Level Non-Uniformity Normalized (GLNN), Size-Zone Non-Uniformity (SZN), Size-Zone Non-Uniformity Normalized (SZNN), Zone Percentage (ZP), Gray-level variance (GLV), Zone variance (ZV), Zone entropy (ZE), Low Gray-Level Zone Emphasis (LGLZE), High Gray-Level Zone Emphasis (HGLZE), Small Area Low Gray-Level Emphasis(SALGLE), Small Area High Gray-Level Emphasis(SAHGLE), Large Area Low Gray-Level Emphasis(LALGLE), Large Area High Gray-Level Emphasis (LAHGLE).

Gray-level dependence matrix (GLDM):

Small Dependence Emphasis (SDE), Large Dependence Emphasis (LDE), Gray-Level Non-Uniformity (GLN), Dependence Non-Uniformity (DN), Dependence Non-Uniformity Normalized (DNN), Gray-level variance (GLV), Dependence variance (DV), Dependence entropy (DE), Low Gray-Level Emphasis (LGLE), High Gray-Level Emphasis (HGLE), Small Dependence Low Gray-Level Emphasis (SDLGLE), Small Dependence High Gray-Level Emphasis (SDHGLE), Large Dependence Low Gray-Level Emphasis (LDLGLE), Large Dependence High Gray-Level Emphasis (LDHGLE).

Neighboring gray tone difference matrix (NGTDM):

Coarseness, Contrast, Busyness, Complexity, Strength.

Part B. Machine learning algorithm

Random Forest

Random forest consists of multiple decision trees. Random forest is a discriminant model that supports both classification and regression problems, and supports multiple classification problems. It is a nonlinear model.

For classification problems, a test sample will be sent to each decision tree for prediction, and then voted, the category with the most votes is the final classification result. For regression problems, the predicted output of the random forest is the average of all decision tree outputs.

In addition, when performing node segmentation in the process of building a tree, the selected segmentation point is no longer the best segmentation point among all features, but the optimal segmentation point in a random subset of features. Due to this randomness, the deviation of the forest usually increases slightly (relative to the deviation of a single non-random tree), but because the average is taken, its variance will also decrease, and it can usually compensate for the increase in deviation, resulting in an overall better model.

Max-relevance and min-redundancy (mRMR)

mRMR is to find a set of features that have the greatest correlation with the final output result in the original feature set(Max-Relevance), but have the smallest correlation between features(Min-Redundancy). The goal is to find a feature subset S with m$\{x_i\}$features.

Max-Relevance: maxD(S,c),D=$\frac{1}{|S|}\Sigma_{xi} \in S^{I(xi;c)}$

Min-Redundancy: minR(S),R=$\frac{1}{|S|2}$ $\Sigma x_i,x_j \in S^{I(xi;xj)}$

Note: x is the feature, c is the categorical variable, and S is the feature subset

Logistics Regression

Logistic regression is a linear regression model, which assumes that the data obey Bernoulli distribution. By means of maximum likelihood function, the gradient descent method is used to solve the parameters, so as to achieve the purpose of dichotomy.LR model can be considered as a linear regression model normalized by Sigmoid function (Logistic equation). Sigmoid compresses the data (LR, middle finger, output y) between [0, 1] and passes through an important point (0, 0.5). In this way, the output is compressed between [0,1], with 0.5 as the boundary value, 0.5 greater than 0.5 as one class, and 0.5 less than 0.5 as another class.

Support vector machine recursive feature elimination (SVM-RFE)

SVM-RFE is a sequential backward selection algorithm based on the maximum interval principle of SVM. It trains the model samples, then sorts the scores of each feature, removes the feature with the minimum score, then trains the model again with the remaining features, carries out the next iteration, and finally selects the required feature number.

Decision tree

Decision tree is an unsupervised learning method for classification and regression. The goal is to create a model that learns simple decision rules from data features to predict the value of an objective variable. The decision tree logically exists as a tree, containing root nodes, internal nodes, and leaf node. Root node: A collection of all the data in a dataset. Internal node: Each internal node is a judgment condition and contains a collection of data in the dataset that satisfies all conditions from the root node to the node. According to the test results of the judgment condition of the internal node, the data set corresponding to the internal node is divided into two or more child nodes. Leaf node: The leaf node is the final category, and the data contained in the leaf node belongs to this category.

Naïve Bayes

Naive Bayesian methods are a series of supervised learning methods based on the assumption that the application of Bayesian theory is naive and that each pair of characteristics is independent of each other. Naïve Bayes formula is as follows:

$$P(Y|X)=\frac{P(X|Y)P(Y)}{P(X)}$$

The main ideas of the Naive Bayes classifier: Through joint probability modeling, theorem of Bayes is used to solve the posterior probability; the category corresponding to the one with the largest posterior probability is used as the prediction category.

k-nearest neighbour (KNN)

KNN is a basic classification and regression method. The input of KNN is the test data and training sample data set, and the output is the category of the test sample. KNN does not show the training process. During the test, the distance between the test sample and all training samples is calculated, and the prediction is made by majority voting according to the category of the nearest K training samples. The algorithm is described as follows:

Input: training data set $T=\{(x_1,y_1),(x_2,y_2),...,(x_n,y_n)\}$ and test data x.

$x_i \in R^n, y_i \in \{c_1,c_2,...,c_K\}, y_i \in \{c_1,c_2,...,c_K\}$

Output: the category to which instance x belongs.

According to the given distance metric, find the k samples closest to x in the training set T. The neighborhood of x covering these k points is recorded as $N_k(x)$. In $N_k(x)$, according to the classification rules (such as Majority vote) determines the category y of x:

$y=\arg\max_j \sum x_i \in N_k(x) I\{y_i=c_j\}, i=1,2,...,n; j=1,2,...,K$

Support vector machine (SVM)

Mainly used to solve data classification problems in the field of pattern recognition, which belongs to a kind of supervised learning algorithm. It has strong nonlinear classification ability, showing many unique advantages in solving small sample, nonlinear and high-dimensional pattern recognition problems.

Bagging

A learning algorithm may have larger model deviations on multiple different training data sets from the same distribution, that is, the model has a larger variance. To solve this problem, the output of multiple models can be synthesized. The regression problem can be averaged, and the

majority problem can be used for classification problems. The above is the core idea of Bagging.

The Bagging algorithm is to perform N Bootstrap sampling on the training data set to obtain N training data subsets, and use the same algorithm to establish decision trees for each subset. The final classification result is the majority vote of the results of N decision trees.

Extremely randomized trees

Similar points to random forest: 1. bootstrap a sample. 2. Randomly select some features to construct a tree. We know that random forests are composed of many random decision trees, and extremely randomized trees are more random than random forests.

Differences from random forests: each decision tree selects points in different ways. For ordinary decision trees, each feature is divided according to a certain standard (information gain or gini impure), and in extremely randomized trees, the choice of division points is more random.

AdaBoost

Given a training data set: $(x_1, y_1),...,(x_n,y_n)$, $y_i \in \{1,-1\}$ is used to represent the category label of the training sample, i=1,...,N.
The purpose of Adaboost is to learn a series of weak classifiers or basic classifiers from training data, and then combine these weak classifiers into a strong classifier.

Gradient boosting tree

It is an algorithm that classifies or regresses data by using an additive model (that is, a linear combination of basic functions) and continuously reducing the residuals generated during the training process. The advantages of GBDT are as follows:

- Natural processing of mixed data (heterogeneous features)
- Strong predictive power
- Robustness to outliers in the output space (implemented by a robust loss function)

Least absolute shrinkage and selection operator (LASSO)

Lasso regression is also called L1 regularization of linear regression. Lasso regression makes some coefficients smaller, and even some coefficients with smaller absolute values directly become 0. Therefore, it is especially suitable for parameter reduction and parameter selection, so it is used to estimate sparseness Linear model of parameters. It obtains a more refined model by constructing a penalty function, which makes it compress some coefficients and set some coefficients to zero. Therefore, the advantage of subset shrinkage is retained, and it is a biased estimate for processing data with multicollinearity.
